# Aggregative Adherence and Intestinal Colonization by Enteroaggregative *Escherichia coli* Are Produced by Interactions among Multiple Surface Factors

**DOI:** 10.1128/mSphere.00078-18

**Published:** 2018-03-21

**Authors:** Laura V. Blanton, Lawrence T. Wang, Jennifer Hofmann, Joshua DuBow, Alexander Lafrance, Stephen Kwak, Levi Bowers, Mandara A. Levine, Charles O. Hale, Philip M. Meneely, Iruka N. Okeke

**Affiliations:** a Department of Biology, Haverford College, Haverford, Pennsylvania, USA; b Department of Pharmaceutical Microbiology, Faculty of Pharmacy, University of Ibadan, Ibadan, Oyo State, Nigeria; University of Rochester

**Keywords:** enteroaggregative *Escherichia coli*, adherence, aggregative adherence fimbriae, antiaggregative protein, diarrhea, diarrheagenic *Escherichia coli*, dispersin, heat-resistant agglutinin 1, intestinal colonization

## Abstract

Enteroaggregative *Escherichia coli* (EAEC) bacteria are exceptional colonizers that are associated with diarrhea. The genome of EAEC strain 042, a diarrheal pathogen validated in a human challenge study, encodes multiple colonization factors. Notable among them are aggregative adherence fimbriae (AAF/II) and a secreted antiaggregation protein (Aap). Deletion of *aap* is known to increase adherence, autoaggregation, and biofilm formation, so it was proposed that Aap counteracts AAF/II-mediated interactions. We hypothesized that Aap sterically masks heat-resistant agglutinin 1 (Hra1), an integral outer membrane protein recently identified as an accessory colonization factor. We propose that this masking accounts for reduced *in vivo* colonization upon *hra1* deletion and yet no colonization-associated phenotypes when *hra1* is deleted *in vitro*. Using single and double mutants of *hra1*, *aap*, and the AAF/II structural protein gene *aafA*, we demonstrated that increased adherence in *aap* mutants occurs even when AAF/II proteins are genetically or chemically removed. Deletion of *hra1* together with *aap* abolishes the hyperadherence phenotype, demonstrating that Aap indeed masks Hra1. The presence of all three colonization factors, however, is necessary for optimal colonization and for rapidly building stacked-brick patterns on slides and cultured monolayers, the signature EAEC phenotype. Altogether, our data demonstrate that Aap serves to mask nonstructural adhesins such as Hra1 and that optimal colonization by EAEC is mediated through interactions among multiple surface factors.

**IMPORTANCE** Enteroaggregative *Escherichia coli* (EAEC) bacteria are exceptional colonizers of the human intestine and can cause diarrhea. Compared to other *E. coli* pathogens, little is known about the genes and pathogenic mechanisms that differentiate EAEC from harmless commensal *E. coli*. EAEC bacteria attach via multiple proteins and structures, including long appendages produced by assembling molecules of AafA and a short surface protein called Hra1. EAEC also secretes an antiadherence protein (Aap; also known as dispersin) which remains loosely attached to the cell surface. This report shows that dispersin covers Hra1 such that the adhesive properties of EAEC seen in the laboratory are largely produced by AafA structures. When the bacteria colonize worms, dispersin is sloughed off, or otherwise removed, such that Hra1-mediated adherence occurs. All three factors are required for optimal colonization, as well as to produce the signature EAEC stacked-brick adherence pattern. Interplay among multiple colonization factors may be an essential feature of exceptional colonizers.

## Abstract (precis)

Enteroaggregative *Escherichia coli* (EAEC) bacteria are exceptional colonizers of the human intestine and can cause diarrhea. Compared to other *E. coli* pathogens, little is known about the genes and pathogenic mechanisms that differentiate EAEC from harmless commensal *E. coli*. EAEC bacteria attach via multiple proteins and structures, including long appendages produced by assembling molecules of AafA and a short surface protein called Hra1. EAEC also secretes an antiadherence protein (Aap; also known as dispersin) which remains loosely attached to the cell surface. This report shows that dispersin covers Hra1 such that the adhesive properties of EAEC seen in the laboratory are largely produced by AafA structures. When the bacteria colonize worms, dispersin is sloughed off, or otherwise removed, such that Hra1-mediated adherence occurs. All three factors are required for optimal colonization, as well as to produce the signature EAEC stacked-brick adherence pattern. Interplay among multiple colonization factors may be an essential feature of exceptional colonizers.

## INTRODUCTION

Enteroaggregative *Escherichia coli* (EAEC) bacteria are diarrhea-associated *E. coli* strains that adhere to epithelial cells in a characteristic “stacked-brick” or aggregative formation. EAEC bacteria are exceptional colonizers that also adhere to one another and to solid support materials, including glass. They form copious biofilms *in vitro* and *in vivo* and have been associated with persistent diarrhea. EAEC accounts for a large but imprecisely estimated fraction of the burden from diarrheal diseases, particularly in developing countries (1, 2). However, with difficulties in EAEC detection and with pathogenesis remaining largely understudied, EAEC bacteria are among the most neglected bacterial pathotypes (3).

EAEC possesses a large and diverse repertoire of adhesins. There are at least five different types of EAEC-specific fimbriae, termed aggregative adherence fimbriae (AAF). Specific EAEC strains can also express other *E. coli* pili, such as ECP, pSERB, and type I fimbriae (4). They may also carry nonstructural factors, including MAM7; one or more alleles of antigen 43; other autotransporter adhesins; one or more agglutinins (including Hra1/Hek, Hra2, and Tia); and Shf (5–12). It is almost certain that other EAEC adhesins remain to be characterized. In addition to adhesins, EAEC colonization is enabled by flagellin, secreted protein autotransporters, and an antiadherence protein (Aap; also known as dispersin) (13–16). Aap is exported from the cell by the Aat secretion system and forms a loosely associated coat on the cell surface (17). EAEC strains differ in the number and repertoire of colonization factors in their genomes (18, 19).

Quite a few EAEC colonization factors have been studied in some detail but almost always in isolation. In this study, we sought to identify and understand interactions among AAF/II, Aap, and Hra1 in prototypical EAEC strain 042, for which a reference genome is available (5). AAF/II, the first 042 colonization factor described, has been significantly associated with diarrheal disease in some studies (19, 20). Aap and Hra1 are each found in a wide variety of *E. coli* isolates, but, among fecal *E. coli* isolates, they are most prevalent among EAEC strains (6, 21). Hra1 appears to function as an accessory and autoadhesion factor, while Aap-mediated antiaggregation appears to be a critical factor in ensuring that hyperadhesive EAEC colonizes mucosal surfaces optimally (13, 22).

Our study was prompted by the earlier finding that Hra1 is sufficient to produce colonization-associated phenotypes *in vitro* but is not required for these phenotypes in 042 (23). Nonetheless, as we have previously reported, Hra1 is required for optimal production of the EAEC-defining aggregative adherence pattern as well as for optimal colonization in the *Caenorhabditis elegans* slow-kill assay (23). In this study, we showed that Aap, reported earlier to produce antiadherence by interfering with AAF/II, is implicated in ameliorating Hra1-mediated adherence.

The *hra1* gene is located on the chromosome of EAEC 042, while AAF/II and Aap are both encoded on a large virulence plasmid that contains other adhesin and virulence genes. As with the *aaf* genes, *hra1* is sufficient to confer autoaggregation, biofilm formation, and EAEC-defining aggregative adherence to laboratory *E. coli* strains (22–24). Interestingly, removal of *hra1* does not quantitatively affect autoaggregation, biofilm production, or adherence, suggesting that Hra1 is redundant or is masked *in vitro*. However, Hra1 appears to be unmasked *in vivo*, as deletion of *hra1* impairs *C. elegans* colonization. The *hra1* mutant also displays qualitative differences in adherence, in that the characteristic stacked-brick adherence pattern on human epithelial cells (HEp2) is disarrayed (23). When *hra1* mutants are complemented, the stacked-brick adherence pattern on HEp2 cells and *C. elegans* killing rates are restored to those seen with the wild-type strain (23). Earlier work showed that deletion of *aafA* obliterates colonization-associated phenotypes, even though the evaluated mutants must have carried the *hra1* gene (24). These phenotypes point to the possibility that Hra1 might be sterically masked *in vitro*, such that its effects are seen only when the masking agent is removed at critical points *in vivo*. Masking of nonfimbrial adhesins has been described for other adhesins and pathogens (25, 26). Identification of factors masking the *hra1* phenotype not only furthers functional characterization of Hra1 but also increases our understanding of interplay between other EAEC colonization factors and Hra1.

## RESULTS AND DISCUSSION

### Hra1 is masked *in vitro*, but physical removal of AAF/II does not unmask Hra1.

**FIG 1:**
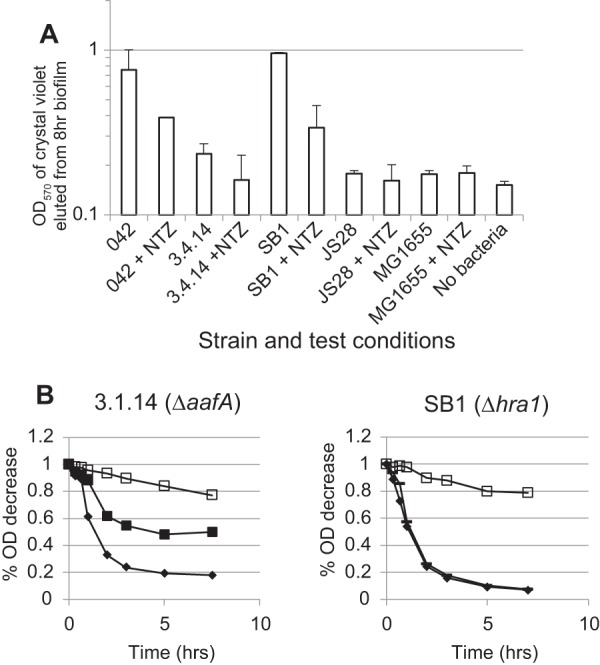
AAF/II does not mask colonization-associated phenotypes produced by Hra1. (A) Biofilm formation of EAEC strain 042 and its *hra1* and *aafA* mutants (SB1 and 3.4.14, respectively) and of *aggR* mutant JS28 in high-glucose DMEM at 8 h in the presence and absence of AAF/II fimbrial assembly inhibitor NTZ. Biofilm formation was measured on a logarithmic scale as the absorbance at 570 nm of crystal violet eluted from a stained and fixed biofilm. With the exception of *hra1* mutant SB1, all of the tested mutants produced significantly less-copious biofilm than 042 (*P* < 0.01). NTZ significantly reduced biofilm formation by 042 and SB1 (*P* < 0.02) but not 3.4.14, JS28, or negative-control *E. coli* K-12 strain MG1655. (B) Autoaggregation of 042 (solid diamonds), SB1 (*Δhra1*; crossed lines), 3.4.14 (*ΔaafA*; solid squares), and MG1655 (K-12 control; open squares) in high-glucose DMEM.

The finding that Hra1 is able to confer autoaggregation and adherence *in vitro* but that EAEC 042 *hra1* mutants are not deficient in these phenotypes suggested that Hra1 might be sterically masked *in vitro* (23). Because much of the literature points to fimbrial masking of short adhesins (26, 27), we first investigated a possible role of AAF/II in masking Hra1. Shamir et al. demonstrated that nitazoxanide (NTZ) blocks the assembly of AAF/II (28). Addition of NTZ to bacterial cultures of wild-type EAEC 042 or of its isogenic *hra1* mutant SB1 reduced biofilm formation to similar extents in the two strains (Fig. 1A). This suggests that while AAF/II does contribute to biofilm formation, removal of AAF/II was not sufficient to unmask Hra1’s contribution to biofilm formation. We tested 042 *aafA* mutant 3.4.14 (24) and found that it is deficient in *in vitro* biofilm formation, consistent with the model that removal of AAF/II fimbriae does not unmask Hra1 or other nonstructural factors contributing to this phenotype (and providing an explanation for why deletion of *aafA* in 1997 [24] did not spur the search for other EAEC adhesins). In concurrence with the biofilm formation data, whilst 042 and SB1 autoaggregated to similar extents, 3.4.14 was less able to autoaggregate (Fig. 1B). Altogether, the data demonstrate that AAF/II does not mask the adhesive properties of Hra1.

### Aap masks Hra1 *in vitro* autoaggregation and biofilm formation.

**FIG 2:**
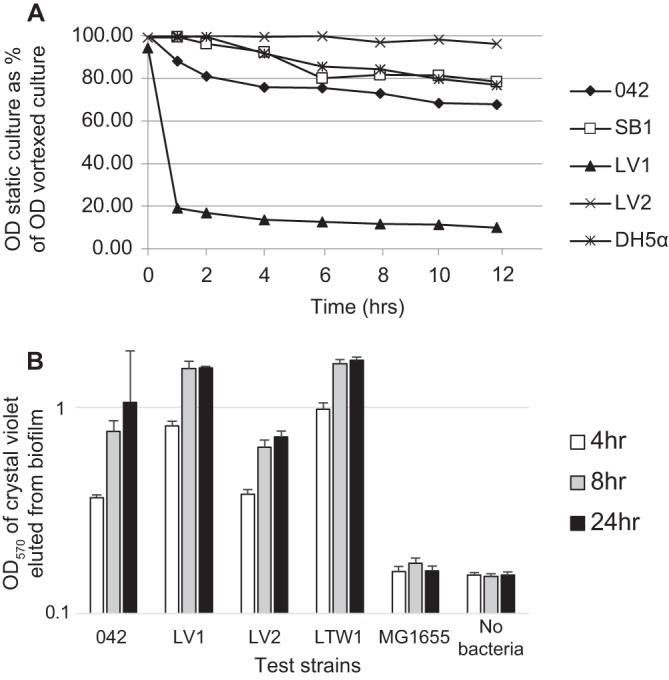
Phenotypes of double mutants suggest that Aap masks Hra1. (A) Autoaggregation by 042, *hra1* mutant SB1, and *aap* deletion mutants LV1 (Δ*aap*), LV2 (Δ*aap* Δ*hra1*), and LTW1 (Δ*aap* Δ*aafA*) in LB. The data are shown as OD_600_ measured at the top of a static culture compared to that measured at the top of a parallel culture that was subjected to vortex mixing. (B) Biofilm formation at 4, 8, and 24 h by 042 and its *aap* deletion mutants LV1 (Δ*aap*), LV2 (Δ*aap* Δ*hra1*), and LTW1 (Δ*aap* Δ*aafA*). *E. coli* K-12 strain MG1655 was a negative control. Biofilm formation was significantly higher than that seen with 042 in LV1 and LTW1 at 8 h (*P* < 0.05). At 24 h, biofilm formation by double *aap hra1* mutant LV2 was significantly lower than that by *aap* mutant LV1 and by *aap aafA* mutant LTW1 (*P* < 0.01).

Deletion of *aap*, which encodes the EAEC antiaggregation protein also known as dispersin, leads to increased autoaggregation and adherence of EAEC strain 042. The increased autoaggregation and adherence have previously been hypothesized to be due to interference with AAF/II (13, 29). We deleted the *aap* gene alone (mutant strain LV1), as well as in combination with *hra1* (mutant strain LV2), from 042. As shown in Fig. 2, and in agreement with studies of other *aap* mutants in the literature (13), *aap* mutant LV1 had increased autoaggregation relative to the wild-type 042 strain. In contrast, the *aap hra1* double mutant LV2 was deficient in autoaggregation (Fig. 2A). *In vitro* biofilm formation experiments revealed that *aap* mutant LV1 formed denser biofilms than 042, particularly at early time points, but that *hra1 aap* mutant LV2 did not (Fig. 2B).

### Aap’s role in colonization is associated only minimally with interactions with AAF/II.

**FIG 3:**
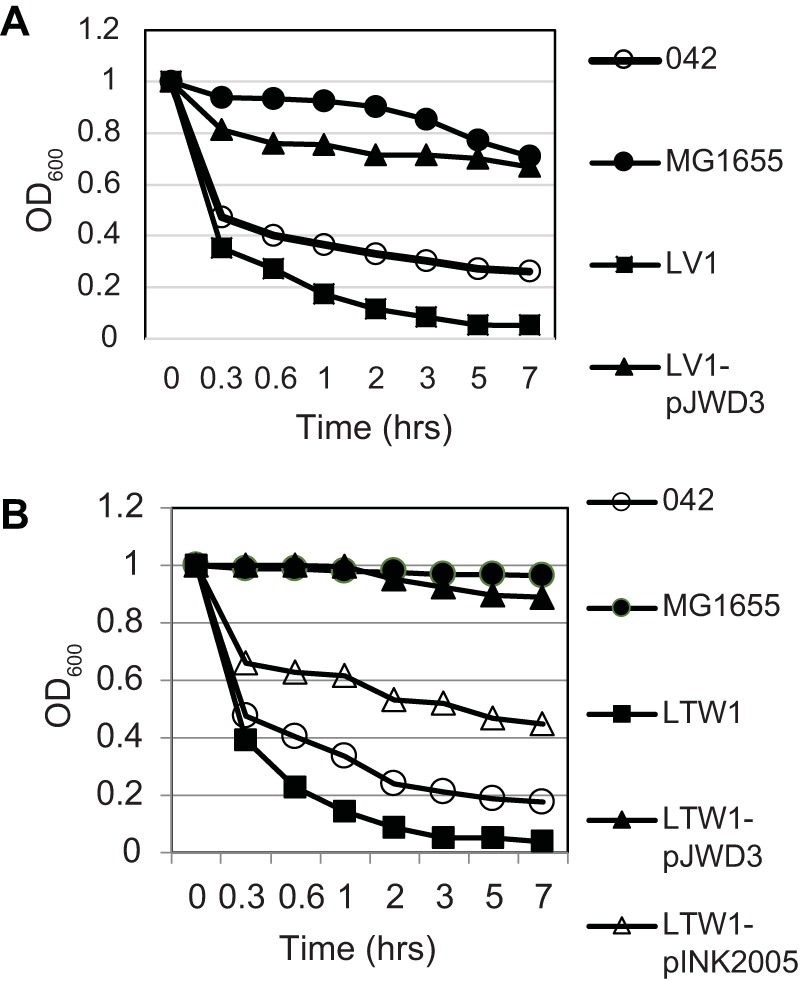
Autoaggregation of *aap aafA* double mutant LTW1 and its *aap* (pJWD3) and *aafA* (pINK2005) transcomplements. The data are absolute OD_600_ values measured at the top of a static culture of each test or control strain.

Though these data indicated that removal of Aap unmasks Hra1, hyperaggregation with the deletion of *aap* had previously been attributed to interactions with AAF/II (13, 29). To better understand interactions of Aap with AAF/II, we constructed and tested *aap aafA* double mutant strain LTW1. LTW1 had biofilm-forming abilities that were quantitatively similar to those seen with *aap* mutant LV1 (Fig. 2B), suggesting that unmasking of Hra1 is the principal basis for the enhanced biofilm formation of *aap* mutants (13). In autoaggregation assays, SB1 showed no defect and 3.4.14 showed reduced autoaggregation (Fig. 1B). As shown in Fig. 3, both LV1 and *aap aafA* double mutant LTW1 autoaggregated more than the wild-type strain. When the LTW1 mutant was complemented with the *aafA* gene, autoaggregation was reduced compared to both the LTW1 mutant and the wild type, supporting the idea that Aap masks Hra1 (Fig. 3B). This finding suggests that the presence of *aap* may be necessary for optimal autoaggregation by Hra1 and/or AAF/II.

**FIG 4:**
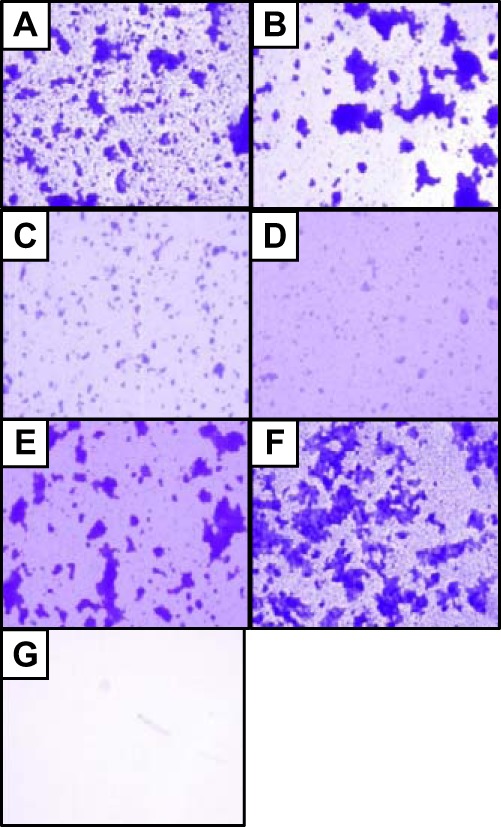
Fixed and stained 24-h biofilms produced on vertically mounted slides by bacteria cultured in high-glucose DMEM (×400 magnification). (A) Wild-type EAEC 042. (B) LV1 (Δ*aap*). (C) SB1 (Δ*hra1*). (D) 3.4.14 (Δ*aafA*). (E) LV2 (Δ*aap* Δ*hra1*). (F) LTW1 (Δ*aap* Δ*aafA*). (G) *E. coli* K-12 MG1655.

As biofilm formation is a complex phenotype dependent on autoaggregation, solid-surface adherence, and other properties, we sought to observe physical differences in biofilms produced by 042 and the *hra1*, *aafA*, and *aap* knockout mutants qualitatively. Biofilms produced by *aap* mutants had morphologies (discernible by light microscopy after fixing and staining) that were distinctly different from those exhibited by other biofilm-forming strains. These morphologies were visible in biofilms grown in multiwell plates (data not shown) and were even more pronounced in biofilms cultured on vertically mounted slides (Fig. 4). Wild-type EAEC 042 produced a mix of single cells, small aggregates, and larger microcolonies, while deletion of *aap* led to a predominance of large microcolonies and clearing of the single cells and small aggregates. Deleting either of the *hra1* and *aafA* adhesin genes reduced overall adherence and abolished large-microcolony formation; however, the effects produced by deleting *hra1* were not identical to those seen by deleting *aafA*. Three-dimensional aggregates were greater in number and size in all strains possessing *hra1* than in strains lacking *hra1* (Fig. 4). Double knockout *aap hra1* and *aap aafA* strains showed phenotypes more similar to the wild-type phenotype than to those seen with any of the single mutants.

**FIG 5:**
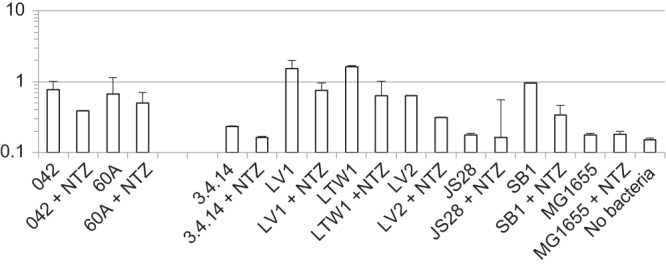
Effect of NTZ on biofilm formation by EAEC strains 042 and 60A and genetic derivatives of AAF/II-producing EAEC strain 042. NTZ significantly reduced biofilm formation by 042, LV1, LV2, SB1, and LTW1 (*P* < 0.02) but not 60A, 042 mutants 3.4.14 and JS28, or negative-control *E. coli* K-12 strain MG1655.

The differences in biofilm structure pointed to early configuration as a likely determinant of eventual biofilm architecture. We therefore examined the initiation of biofilm formation on glass slides over a time course of 3 h. While *aafA* mutant strain 3.1.14 showed considerably less attachment to the slide, the formation of early 3-dimensional aggregates, visible as “knots” on fixed crystal violet-stained slides, was seen only in 042 and its derivatives possessing the *hra1* gene (see Fig. S1 in the supplemental material). Knots were not visible when *hra1* was deleted alone (SB1) or in combination with *aap* (LV2) but could clearly be seen in the *aap aafA* double mutant LTW1. We were unable to construct and select a triple *hra1 aap aaf* mutant, despite multiple attempts to do so. This was made difficult in part by the fact that 042, itself a strain that is challenging to engineer, is resistant to three of the most popular markers for allelic exchange—tetracycline, chloramphenicol, and streptomycin (50)—and three others (kanamycin, trimethoprim, and ampicillin) were used to engineer the successful constructs. As NTZ is an antiadherence agent that has previously been shown to interfere with AAF/II-mediated phenotypes by inhibiting fimbrial assembly (1), we studied the effects of this compound on biofilm formation in EAEC 042 and the mutant strains. In the presence of AAF/II fimbrial assembly inhibitor NTZ, LV1 and LV2, like 042 and SB1, showed reduced biofilm formation, while AAF/II-negative EAEC strain 60A (30) and EAEC 042 *aafA* mutant 3.4.14 did not (Fig. 5). Unexpectedly, NTZ also reduced biofilm formation of LTW1, the *aap aafA* double mutant, suggesting that NTZ inhibits an additional factor other than AAF/II. As 3.4.14 showed very little biofilm formation overall, this unknown factor is not likely to be Aap.

10.1128/mSphere.00078-18.1 FIG S1  Early-stage biofilms from EAEC strain 042 and the test mutants. Biofilm initiation was performed on coverslips (×400 magnification). Biofilms produced by 042 and its mutants and by *E. coli* K-12 control strain MG1655 were fixed and stained 0.5, 1.5, and 3 h after inception to view early-stage biofilm formation. Download FIG S1, TIF file, 0.2 MB. Copyright © 2018 Blanton et al. 2018 Blanton et al. This content is distributed under the terms of the Creative Commons Attribution 4.0 International license.

### Hemagglutination profiles and aggregate formation *in vitro* and *in vivo* are consistent with Aap’s role as a mask for Hra1.

**FIG 6:**
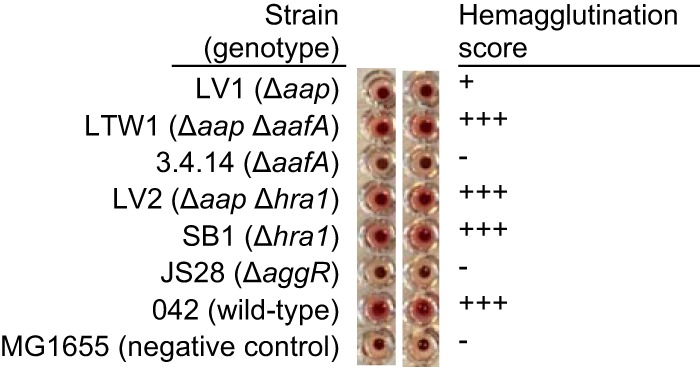
Agglutination of sheep erythrocytes measured after incubation with 042 and mutants in this study. Nonagglutinating erythrocytes sank to the center of the bottom of the well, presenting a well-defined dot. Hemagglutination is indicated by formation of a shield at the bottom of the well as a consequence of immobilization of agglutinated erythrocytes across the whole well.

Hemagglutination results supported the idea of the autoaggregation and biofilm formation phenotypes displayed by 042 and the studied mutants. 042 agglutinated sheep erythrocytes, but an *aafA* mutant 3.4.14 did not (Fig. 6), consistent with previous reports of AAF/II-mediated hemagglutination (24). *aap aafA* double mutant LTW1 produced wild-type hemagglutination, indicating the presence of an additional adhesin(s) unmasked by Aap removal (Fig. 6). The *hra1* gene is sufficient to confer hemagglutination (23); however, deletion of *hra1* in SB1 had no effect on hemagglutination when AAF/II was still present. These data suggest that more copious *in vitro* adherence or autoaggregation may not necessarily translate to improved colonization. We noticed that when *aap* alone was deleted, the mutant LV1 was able to agglutinate sheep erythrocytes but that it did so less well than wild-type EAEC 042 or the *aap aafA* double mutant. Vertical biofilm slides demonstrated that aggregate size is likely modulated by *aap* and the two adhesins and that a mix of small-large aggregates is produced optimally only when Aap, Hra1, and AAF/II are all present (Fig. 4). Formation of larger or denser LV1 aggregates may preclude LV1-erythrocyte interactions and illustrates the need for an adhesin “organizer,” a role that Aap appears to play.

**FIG 7:**
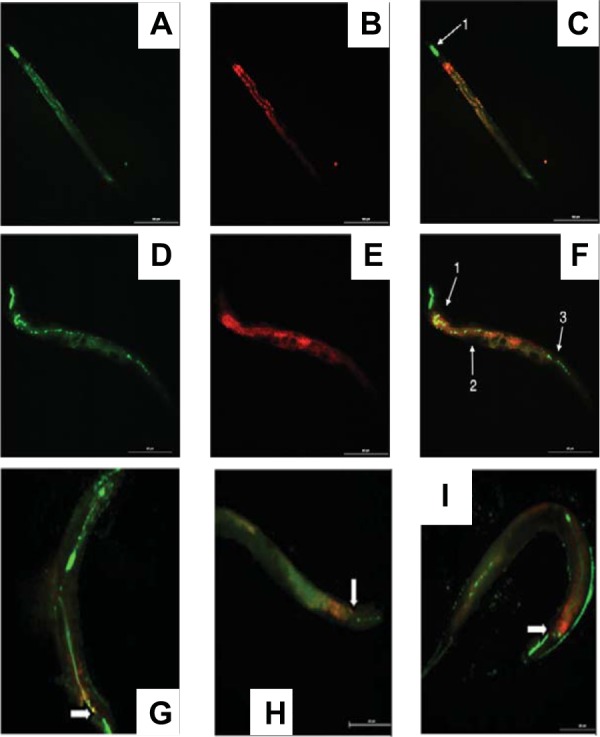
Visualization of GFP-expressing bacteria after they had been ingested by N2 *C. elegans.* Panels A to F show the green channel depicting GFP-expressing bacteria and worm autofluorescence (A and D), the red channel depicting worm autofluorescence alone (B and E), and merging of the two channels (C and F). (A to C) A worm fed with OP50-GFP, with the grinder marked with an arrow. (D to F) Worm fed with 042. Arrows mark the pharynx (1), a location slightly after the anterior deirid (2), and the hindgut region (3) of the worm. Panels G, H, and I show merged images for mutants LV1, LV2, and SB1. White block arrows show the position of the grinder.

We used a previously developed *C. elegans* assay to assess EAEC colonization (31). Worms fed with negative-control *E. coli* strain OP50-GFP (OP50-green fluorescent protein) harbored high densities of bacteria in *C. elegans* buccal cavities and some bacteria beyond the pharynxes but were devoid of visible bacteria by the midguts and beyond (Fig. 7). Additionally, no distinct microcolonies of OP50-GFP could be seen within the intestine of the *C. elegans* fed this strain at ×400 magnification. In contrast, in worms fed with 042-GFP, we observed distinct colonies just distal from the pharynx, slightly after the anterior deirid, and in the hindgut region of the worms (Fig. 7). EAEC microcolonies with an average diameter of 0.94 + 0.37 µm were seen in the three regions that showed 042-GFP colonization. A total of 16 + 5 distinct microcolonies were counted on average throughout the *C. elegans* intestine.

The GFP-expressing *aap* mutant, LV1-GFP, showed a decreased ability to spread within *C. elegans*, as the bacteria traveled no farther than 21% + 21% of the worm’s length (mean + standard error of the mean compared to the 31.6% + 16% traveled by 042; *P* = 0.05) (Fig. 7G). At ×400 magnification, we observed that *C. elegans* fed with LV1-GFP showed distinct EAEC microcolonies with larger diameters than those seen in *C. elegans* fed with 042-GFP (2.19 + 1.71 µm; *P* = 3.44e−7). Thus, removal of Aap increased microcolony size but decreased the length of the worm gut colonized. SB1-GFP, deleted for *hra1*, showed a more dispersed colonization pattern throughout the intestine of the *C. elegans* than 042-GFP, OP50-GFP, or LV1-GFP (Fig. 7I). On average, the length of colonization by SB1 made up 64.0% + 17% of the length of the *C. elegans*, which was significantly different from the colonization seen with 042 (*P* = 0.03). When ×400 magnification was used, we observed that *C. elegans* fed with SB1-GFP did not show distinct EAEC colonies such as were found in *C. elegans* fed with 042-GFP or LV1-GFP.

With LV2-GFP, the *hra1 aap* double mutant, we saw a colonization pattern throughout the intestine of *C. elegans* that was more dispersed than that seen with SB1-GFP (Fig. 7H). In *C. elegans* fed with LV2-GFP, EAEC visibly colonized at a significantly higher level than 042-GFP (77.6% + 8.4% of the length of *C. elegans*; *P* = 0.01). Additionally, also similarly to the results seen with *C. elegans* fed with SB1-GFP, LV2-GFP did not show distinct EAEC colonies such as were seen with 042-GFP or LV1-GFP.

**FIG 8:**
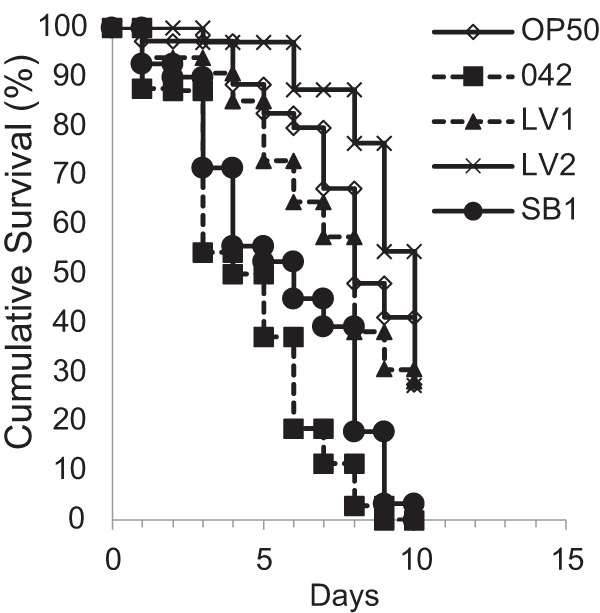
Kaplan-Meier survival curves for *C. elegans* N2 nematodes fed OP50 (negative control), EAEC 042, *hra1* mutant SB1, *aap* mutant LV1, and *hra1 aap* double mutant LV2. The cumulative rates of survival were significantly different between EAEC 042 and OP50, between 042 and SB1, and between 042 and LV1 (*P* < 0.05). Worms fed LV2, however, demonstrated significantly higher cumulative survival rates than were seen with those fed 042 (*P* = 0.005) and OP50 (*P* < 0.05).

These qualitative observations were associated with a significant reduction in *C. elegans* low kill rates for mutants. Survival rates in the slow-kill assay were significantly different between EAEC 042 and mutants SB1 and LV1 (*P* < 0.05). Worms fed LV2 demonstrated even higher cumulative survival rates (*P* = 0.005 compared to 042 results) and LV2 showed less virulence than even negative-control strain OP50 in this assay (Fig. 8). Altogether, the data show that, whilst Hra1 promotes aggregation and Aap promotes dispersion, the *hra1* and *aap* mutants are both deficient in *C. elegans* colonization compared to the wild-type strain. A balance between aggregation and dispersion is essential for optimal colonization; absence of both genes in the LV2 mutant completely attenuates pathogenesis.

### Aggregative adherence to eukaryotic cells requires Aap when AAF/II and Hra1 are both present.

**FIG 9:**
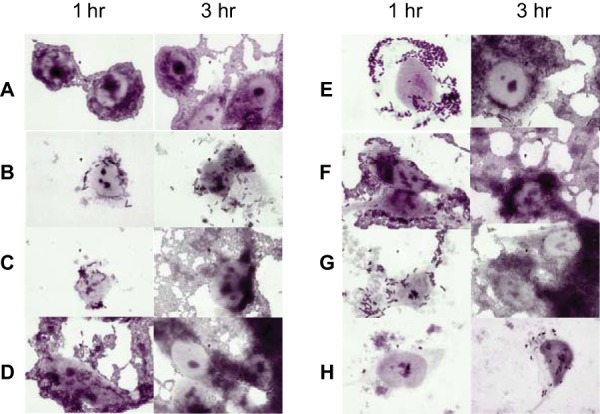
HEp-2 adherence assay of EAEC strain 042 and mutant strains. The adherence of each strain after 1 h and 3 h of incubation of bacteria with cells is visualized. (A) Wild-type EAEC strain 042. (B) Negative-control *E. coli* K-12 strain MG1655. (C) LV1 (Δ*aap*). (D) LTW1 (Δ*aap* Δ*aafA*). (E) 3.4.14 (Δ*aafA*). (F) LV2 (Δ*aap* Δ*hra1*). (G) SB1a (Δ*hra1*). (H) JS28 (Δ*aggR*).

EAEC bacteria are defined by their hallmark stacked-brick adherence pattern on cultured HEp-2 cells. A *hra1* mutant often produces a more disheveled arrangement, a phenotype that can be complemented in *trans* (23), but otherwise, the differences in adherence patterns among the different mutants are at best subtle after 3 h of incubation. As shown in Fig. 9, although the conventional time for the HEp-2 assay is 3 h (32), disheveled adherence is most visible at early time points, such as 1 h after incubation of the bacteria on tissue culture monolayers. After 1 h of incubation, neither the *hra1* mutant nor the *aap* mutant produced a tidy stacked-brick arrangement. In contrast, the *aap hra1* double mutant rapidly adhered in a stacked-brick formation. Additionally, *aafA* mutant 3.4.14 showed weak adherence and the stacked-brick formation at 1 h (Fig. 9E) but the *aap aafA* mutant produced an adherence pattern very similar to that seen with the wild-type strain (Fig. 9D). Thus, when *aap*, *hra1*, or *aafA* is deleted alone, early adherence is less effective and less organized. We hypothesize that Aap contributes to choreographing adhesins in order to permit rapid optimal adherence. The hemagglutination data shown in Fig. 7 agree with this model, as deletion of *aap* alone produced reduced hemagglutination, whereas hemagglutination occurred at wild-type levels when *aap* was deleted together with *aafA* or *hra1*.

### Aap masks other adhesins.

**FIG 10:**
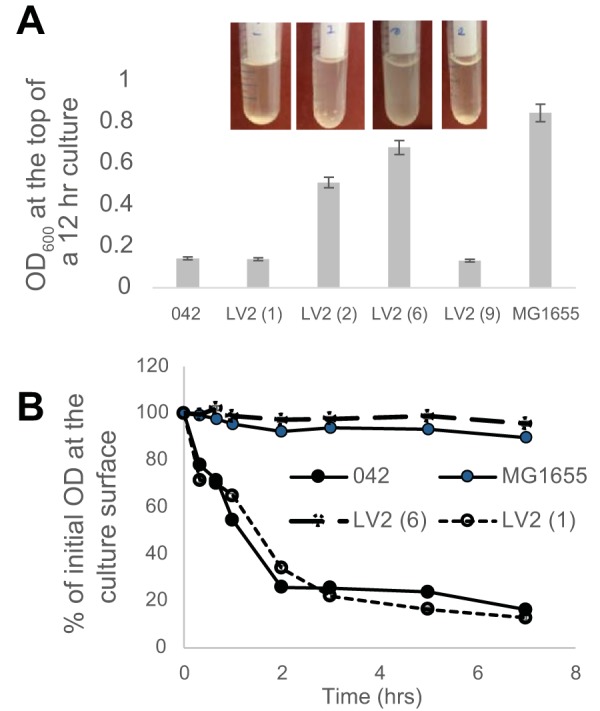
Autoaggregation of selected LV2 colonies suggests that Aap masks an unknown phase-variable factor. (A) Photo of cultures from 4 colonies and OD_600_ data determined from the surface of those colonies at 12 h. (B) Autoaggregation of one autoaggregating colony (culture 1) and one nonautoaggregating colony (culture 6) of LV2. Data were obtained by independently testing two descendants of each colony, and the average decline in OD_600_ of the original culture was plotted. Error bars (generally shorter than the data points) represent standard errors of the means.

While *aap hra1* double mutant LV2 most commonly failed to autoaggregate, we had recorded 4 of 21 experiments in which it did aggregate. We therefore plated out a nonaggregating LV2 culture for single colonies and performed a series of parallel autoaggregation experiments using the 11 isolated colonies thus derived. Eight colonies produced nonaggregating liquid cultures, and three produced highly aggregating cultures; there were no intermediate levels of aggregation. Representatives of both phenotypes are shown in Fig. 10. All aggregating cultures yielded descendants that also aggregated, and nonaggregating cultures yielded nonaggregating descendants in all but one instance. In the one instance where a nonaggregating colony culture had resulted in an aggregating colony, the descendants that were produced from then on retained the aggregating phenotype. Irrespective of the autoaggregation phenotype, all LV2 colonies were genotypically identical. OxyR-mediated switching of any of 042’s antigen 43 alleles could explain this phenotype (33), but it is equally possible that another phase-variable protein is responsible for the switching. While we have yet to identify the specific responsible effector, the results pointed to a potential phase-variable autoagglutinin that is present and unmasked in strain LV2 but whose effects are invisible when Aap is present or when autoaggregation is mediated by Hra1.

### Concluding remarks.

Among the examples of steric masking of short adhesins that are documented in the literature is the masking of self-associating autotransporter proteins antigen 43, AIDA-1, and TibA by fimbriae and capsule (25, 26, 34, 35). Masking is seen with other autotransporters such as EhaA of enterohemorrhagic *E. coli* (36) and SadA, a trimeric *Salmonella* autotransporter masked by O-antigen (37). Masking should be suspected if the presence of a short adhesin is sufficient to confer surface-related phenotypes on heterologous strains but those phenotypes are not obliterated when the gene is deleted from a colonizer. If the masking agent is removed at a critical point *in vivo* but not *in vitro*, the true contribution of the masked factor to colonization and virulence may not be apparent from *in vitro* assays.

Our data from this study point to Hra1 being a principal factor unmasked by Aap but also demonstrate likely interactions with other adhesins. The preexisting view in the literature—that removing Aap results in enhanced adhesive properties of AAF/II fimbriae—relies on the observed collapse of fimbriae onto the bacterial surface in the *aap* mutant (17) and the evidence that an *aap* mutant autoaggregates better than the wild type (13, 17, 29), which we have verified with an independent *aap* mutant. Those data were compiled before our discovery of Hra1 in EAEC. Our new model posits that although both AAF/II and Hra1 are adhesins, the hyperaggregating phenotype of the *aap* mutant is largely attributable to unmasking of Hra1.

Hra1 is required for full colonization *in vivo* (23); our presupposition—strongly supported by the *C. elegans* colonization data—is that although this protein is masked *in vitro*, it is unmasked *in vivo*. Our data open up new issues about the possible unmasking mechanism. One possibility is that fluid flow washes away or otherwise perturbs Aap, which is loosely associated with the cell surface (29), exposing the short self-associating protein and thereby permitting autoaggregation. Sherlock et al. have shown that SAAT proteins TibA and Aida, which are masked by pili (and, in the case of TibA, also by capsule), enhance biofilm formation in continuous flow systems (34, 38). The peristaltic unmasking model is tempting as an explanation for the 042 results, since Aap could accumulate in and around microcolonies until cells detach to form new infection foci. However, it is also possible that other factors, such as a bacterial or host-derived protease, could degrade the masking factor.

This work has demonstrated that Aap most likely exhibits its antiaggregation effect by masking Hra1, rather than by interfering with AAF/II-mediated adherence as previously thought. The presence of Hra1 is sufficient to produce autoaggregation, hemagglutination, thicker biofilms, and even aggregative adherence (23). Our data are consistent with a model wherein the importance of AAF/II in EAEC colonization is derived, at least in part, from the fact that the Hra1 adhesin is masked by Aap. Thus, like many other fimbriae, AAF/II may be critical for initial adherence, but we cannot rule out the possibility of an additional role in longer-term colonization. Removal of Aap, which almost certainly occurs *in vivo*, is necessary to prevent overadherence and to seed new colonization. This, however, also unmasks Hra1, cementing aggregative adherence among cells that remain attached.

Boisen et al. (39) found that combinations of EAEC accessory genomes are more strongly associated with disease than individual factors. The interplay among Hra1, AAF/II, and Aap in 042 is almost certainly relevant to other EAEC strains with loci that encode all three factors or their analogues. It is also a valid consideration for other gut-colonizing bacteria. For example, enterotoxigenic *E. coli* usually expresses at least one of many fimbrial adhesins or colonization factor antigens, including the Hra1 homolog Tia as well as an Aap homolog known as CexE with an associated *aatABCD* secretion system (40). A similar paradigm plays out for the unrelated pathogen *Kingella kingae*, which colonizes the respiratory tract. Optimal *K. kingae* colonization results from interplay between retractable type IV pili, an autotransported outer membrane protein, and a capsule (27). The results of these studies underline the need for in-depth functional analyses of multiple colonization factors in concert. EAEC strains, although highly heterogeneous, typically carry a large number of potential colonization genes and are useful models for arriving at general principles for multifactor colonization.

## MATERIALS AND METHODS

### Bacterial strains, plasmids, and culture conditions.

For maintenance and molecular biology procedures, bacterial strains were routinely cultured in Luria broth (LB) or LB agar and maintained in LB-glycerol (1:1) at −80°C. Antibiotics, including ampicillin (100 µg/ml), chloramphenicol (30 µg/ml), tetracycline (25 µg/ml), and neomycin (50 µg/ml), were added for selection when required. Trimethoprim was used at 200 μg/ml in Mueller-Hinton agar. EAEC strain 042 was originally isolated in Peru and elicited diarrhea in three of five adult volunteers during a human challenge study (41). This and other strains and plasmids used in this study are listed in Table S1 in the supplemental material. We used *E. coli* K-12 strains TOP10 and DH5α (Invitrogen) as host strains for cloned genes unless otherwise indicated. *E. coli* OP50 and genome-sequenced K-12 MG1655 were employed as controls in some experiments. Plasmids with an *oriR6K* origin were maintained in EC100D *pir*-116 (Epicentre) and conjugated to wild-type EAEC strains by the use of *E. coli* SM10λ*pir* as a conjugative donor.

10.1128/mSphere.00078-18.2 TABLE S1  Strains and plasmids used in this work. Download TABLE S1, PDF file, 0.2 MB. Copyright © 2018 Blanton et al. 2018 Blanton et al. This content is distributed under the terms of the Creative Commons Attribution 4.0 International license.

### General molecular biology procedures.

Standard molecular biology procedures were employed (42). DNA was extracted using an Easy-DNA kit (Invitrogen) according to the manufacturer’s instructions. For cloning, DNA was amplified with *Pfx* polymerase (Invitrogen) according to the manufacturer’s instructions. All other amplifications used *Taq*-based PCR Supermix (Invitrogen) and 1 μM oligonucleotide primer in each reaction. Oligonucleotide primer sequences are listed in Table S2. *Taq* amplifications began with a 2-min hot start at 94°C followed by 30 cycles of denaturing at 94°C for 30 s, annealing for 30 s at 5°C below the primer annealing temperature, and extension at 72°C for 1 min for every kilobase of DNA. Ligations were performed using Quick T4 ligase enzyme (NEB), and clones and plasmids were transformed into chemically competent or electrocompetent *E. coli*. Chemically competent and electrocompetent K-12 strains were purchased from Invitrogen and Epicenter. Transformation into EAEC strains was accomplished by electroporation using 2-mm-path-length cuvettes in a Bio-Rad Micropulser, according to the manufacturer’s instructions.

10.1128/mSphere.00078-18.3 TABLE S2  Oligonucleotide primers used in this study. Download TABLE S2, PDF file, 0.1 MB. Copyright © 2018 Blanton et al. 2018 Blanton et al. This content is distributed under the terms of the Creative Commons Attribution 4.0 International license.

### Construction of nonpolar isogenic mutants in strain 042.

Construction and validation of the *hra1* mutant were previously described in detail (23). Other mutants used in this study were analogously prepared by allelic exchange, using the suicide vector pCVD442 (23, 43). Our preliminary assessment revealed that a previously published *aap* mutant had the potential to be polar on heterologous genes (13); therefore, we constructed a new mutant, LV1, in which *aap* is replaced with a *dfrA7* trimethoprim resistance cassette (44). A mutant construct consisting of flanking *aap* sequence marked by a *dfrA7* cassette was produced by first amplifying 1 kb of sequence upstream of the *aap* gene and 1 kb of downstream sequence using primers listed in Table S2, with primers that were just within the *aap* gene carrying XhoI tails. The resulting amplicons were separately cloned into pGEMT (Promega). The downstream flanking region was then excised with XhoI and SalI and ligated into the similarly restricted pGEMT::upstream clone. A *dfrA7* cassette amplified from plasmid pASL01a with primers with XhoI tails was cloned into the XhoI site of the resulting deletion construct. The 2.7-kb trimethoprim selectable deletion construct was then subcloned into pCVD442 to create pLV1. pLV1 was mated into 042 using SM10λpir, and, following allelic exchange, *aap* mutants were selected and verified as previously described (23). A *hra1 aap* double mutant was produced by crossing the *aap* deletion into the *hra1* mutant strain SB1 using pLV1. We additionally crossed this deletion into an *aafA* mutant (24), producing an *aap aafA* double mutant. All mutants were verified by screening for all target genes and pAA bearing the CVD432 locus by PCR, using primers other than those used in construction, detecting the presence of host strain markers *tetA* and *cat* and expression of Hra1 (where applicable). The respective genes cloned into pBR322 were used to complement mutations.

### Autoaggregation.

Bacterial autoaggregation was quantified as bacterial settling rates over time in test media as described previously (23, 26). Overnight cultures of each strain were adjusted to the same optical density at 600 nm (OD_600_). A 5-ml volume of each adjusted culture was placed into each of two separate tubes. One tube remained static, and the other was subjected to vortex mixing before each OD measurement. The tubes were incubated without shaking at 37°C. At designated time points, 0.5 ml was removed from within 2 cm of the surface of the culture, and the OD_600_ was measured. This assay was performed using both LB medium and high-glucose Dulbecco’s minimum essential medium (DMEM) (Invitrogen).

### Biofilm formation.

Bacterial biofilms were built in high-glucose DMEM and then fixed and subjected to crystal violet staining for visualization or quantification following stain elution performed as described by Sheikh et al. (45). We performed biofilm tests using multiwell plates in the traditional assay as well as on vertically mounted slides incubated in tubes in order to visualize biofilms that were built on a vertical plane and that were therefore less biased by gravitational settling.

For the multiwell plate assays, 10 μl of overnight culture was added to 1 ml of test medium in a 24-well plate. Plates were incubated statically, or with rocking, at 37°C. At designated time points, culture medium was aspirated and each well was washed three times with phosphate-buffered saline (PBS) and fixed for 10 min with 75% ethanol. The wells were allowed to dry completely. Fixed biofilms were visualized by staining with 0.5% crystal violet for 10 min, washing with water, and viewing with a stereoscopic zoom microscope. For biofilm quantification, crystal violet was eluted with 1 ml of a 3:1 blend of ethanol-acetone. The OD_570_ of the eluted crystal violet was measured. Quantitation was performed after growth in high-glucose DMEM, which is optimal for biofilm formation by EAEC strain 042 (45). Where required, 0.5 μl of 10 mg/ml of nitazoxanide (NTZ), diluted in dimethyl sulfoxide (DMSO), was used to inhibit AAF/II fimbrial assembly in biofilm experiments. Data were analyzed by an unpaired Student’s *t* test.

For vertical biofilms, 35 ml of medium was added to each sterilized slide in a 50-ml conical tube and 35 µl of overnight bacterial culture was used to seed the biofilm. After incubation, slides were washed, fixed, and stained as described for multiwall plates, and images were taken at ×100 magnification under conditions of oil immersion.

### Eukaryotic cell adherence assays.

Tissue culture cell lines were purchased from the American Type Culture Collection and maintained according to ATCC guidelines. The HEp-2 adherence assay originally described by Cravioto et al. (46) was used with modifications for delineating aggregative adherence (47). HEp-2 cell monolayers were cultured overnight in 8-well chamber slides (for qualitative tests) to 50% confluence in high-glucose DMEM with fetal bovine serum, streptomycin, and penicillin (Invitrogen). Bacteria were cultured in LB broth without shaking at 37°C overnight. On the day of the adherence assay, the HEp-2 cells were washed three times with PBS. Growth medium was replaced with high-glucose DMEM containing 1% mannose (without fetal bovine serum and antibiotics). At 3 h (according to the standard test) or 1 h (in an adapted test to visualize early-stage adherence), culture medium was aspirated and each well was washed three times with PBS. The cells were fixed for 20 min with 70% methanol and then stained for 20 min with a 1:40 dilution of Giemsa stain-PBS. Adherence patterns were observed using oil immersion light microscopy at ×1,000 magnification.

### *C. elegans* slow-kill assay.

We used a slow-kill assay adapted from Aballay et al. (48) and Tan et al. (49) as optimized for EAEC (31) to measure colonization of *C. elegans*. Briefly, 10 hermaphroditic N2 worms at the L4 stage were seeded onto plates of nematode growth medium (NGM) agar 24 h after they had been surface inoculated with test or control bacterial cultures. Worms were transferred to fresh bacterial lawns every 48 h to distinguish the generations. Survival was documented every 24 h, and worms were considered dead when they no longer responded to touch. Strains 042, which has been previously shown to kill *C. elegans* (23, 31), and *E. coli* OP50 were used as positive and negative controls, respectively, and each independent assay was repeated in quadruple. Data from the assays were analyzed via nonparametric Kaplan-Meier statistics, and significant differences were inferred from chi-square analyses performed using the PEPI version 4.0 SURVIVAL program.

To examine bacterial colonization in infected worms, test and control bacterial strains were transformed with pGFP. After verifying that this plasmid had been inserted into each of the EAEC strains and that GFP was successfully produced, the strains were fed to *C. elegans* for 3 days at room temperature on NGM medium. Individual worms were washed with M9 solution in round-bottomed 96-well plates and then placed on microscope slides with a thin layer of 0.8% agarose. *C. elegans* worms were examined under ×100, ×200, and ×400 magnification on a Nikon confocal C1 microscope under conditions of 515-nm and 530-nm wavelength and 605-nm and 675-nm wavelength excitation to examine the degree of colonization and the colonization patterns inside *C. elegans*.
