# Supplementary figures and images for: Aggregative Adherence and Intestinal Colonization by Enteroaggregative Escherichia coli Are Produced by Interactions among Multiple Surface Factors
Source: mSphere. 2018 Mar 21;3(2):e00078-18. doi: 10.1128/mSphere.00078-18 (PMC5863034; doi:10.1128/mSphere.00078-18)

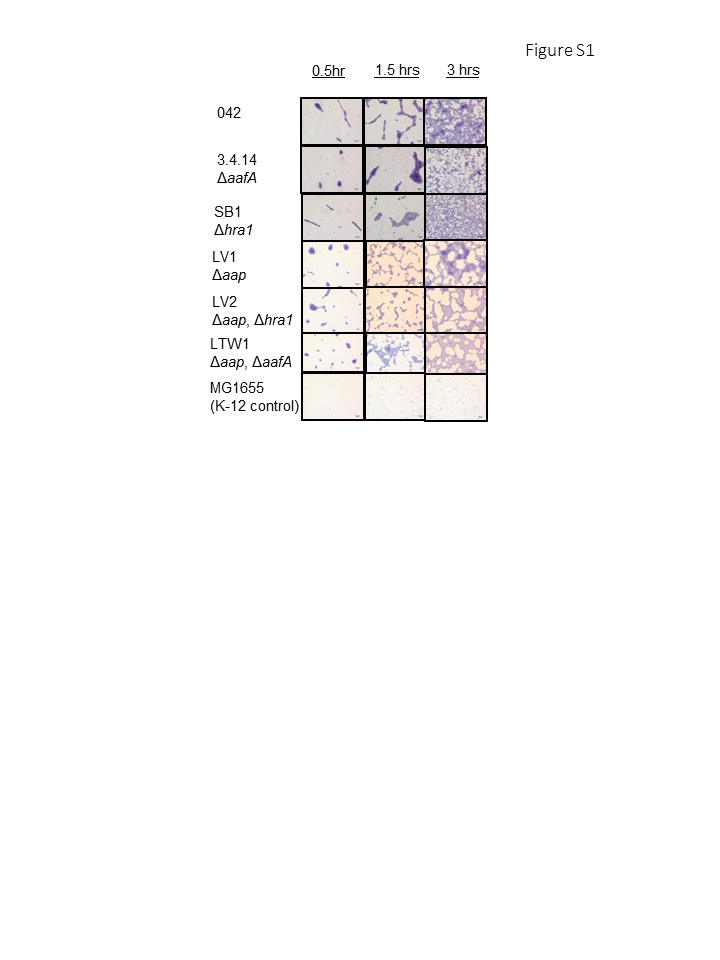

Supplement: FIG S1 [file sph002182496sf1.tif]
