# Supplementary material for: Aggregative Adherence and Intestinal Colonization by Enteroaggregative Escherichia coli Are Produced by Interactions among Multiple Surface Factors
Source: mSphere. 2018 Mar 21;3(2):e00078-18. doi: 10.1128/mSphere.00078-18 (PMC5863034; doi:10.1128/mSphere.00078-18)
Supplement: TABLE S1 [file sph002182496st1.pdf]

Supplemental Table 1. Strains and plasmids used in this work.

**Strains/ plasmids**	**Description or genotype**	**Antibiotic resistance[a]**	**Reference/ Source**
**<u>Strains</u>**			
042	EAEC wild-type O44:H18 isolate from Peru	Sm, Tet, Cm	[1, 2]
60A	EAEC isolate from Mexico, which does not express AAF/II or Hra1 and instead harbors genes encoding AAF/I and Hra2	Amp	[3, 4]
MG1655	*E. coli* K-12 strain used as a negative control in HEp-2 adherence assays	-	*E. coli* stock center [5]
EC100D™ *pir*-116	Host strain for *ori6K* plasmids	-	Epicentre [6]
SB1	042Δ*hra1* isogenic mutant. *hra1::aphA-3*	Km, Sm, Tet, Cm	[7]
LV1	042Δ*aap* isogenic mutant. *aap::dfrA7*	Km, Sm, Tet, Cm	This study
LV2	042Δ*aap*Δ*hra1* isogenic mutant. *aap::dfrA7*,	Km, Sm, Tet, Cm,	This study

	hra1::aphA-3	Tp	
3.4.14	042 with inactivating Tn*phoA* insertion in *aafA*	Km, Sm, Tet, Cm	[8]
LTW1	042Δ*aap*Δ*aafA* mutant. *aap::dfrA7*, *aafA::*Tn*phoA*	Km, Sm, Tet, Cm, Tp	This study
SM10 λpir	Donor strain used for conjugal transfer of pSB1 into 042.<br>*thi thr leuB tonA lacY supE recA*::RP4-2-Tc::Mu-Km	Km	[9]
TOP10	F- *mcr*A .(*mrr-hsd*RMS-*mcr*BC) ö80*lac*Z.M15 .*lac*X74 *deo*R *rec*A1 *ara*D139 *Δ*(*ara-leu*)7697 *gal*U *gal*K *rps*L (StrR) *end*A1 *nup*G	-	Invitrogen
DH5α	F- φ80*lac*ZΔM15 Δ(*lac*ZYA-*arg*F) U169 *rec*A1 *end*A1 *hsd*R17 (rk-, mk+) *gal- pho*A *sup*E44 λ- *thi*-1 *gyr*A96 *rel*A1		Invitrogen
OP50	Non pathogenic *E. coli* strain used for *C.*		[10]

	elegans maintenance		
PA14	*Pseudomonas aeruginosa*		[10]
**<u>Plasmids</u>**			
pBJ1	*hra1* cloned into the *Ssp*I and *Sph*I sites of pBR322	Amp	[7]
pJWD3	*aap* cloned into *Sph*1 and *Sal*1 sites of pBR322	Amp	This study
pINK2005	*aafA* cloned into *Sph*1 and *Sal*1 sites of pBR322	Amp	This study
pBR322	Cloning vector	Amp, Tet	New England Biolabs
pCVD442	Suicide vector *oriR6K sacB*	Amp	[11]
pGEMT	TA cloning vector	Amp	Promega
pASL01a	27 Kb Naturally occurring plasmid carrying *dfrA7*	Amp, Tp, Sm, Hg	[12]
pSB1	*aphA-3* cassette and *hra1* flanking regions (*hra1::aphA-3*) in pCVD442	Amp, Km	[7]

pLV1	*dfrA7 cassette* and *aap* flanking regions (*aap::dfrA7*) in pCVD442		This study
pGFP	GFP-encoding ampicillin-resistant plasmid	Amp	Clontech
pUC18K	pUC18 containing an *aphA-3* cassette preceded by translational stop codons in all three reading frames and followed by a consensus ribosomal binding site		[13]

[a]Amp, ampicillin; Km, kanamycin; Sm, streptomycin; Cm, chloramphenicol; Tet, tetracycline; Hg, Mercury

References

1. Nataro JP, Deng Y, Cookson S, Cravioto A, Savarino SJ, Guers LD, Levine MM, Tacket CO: **Heterogeneity of enteroaggregative Escherichia coli virulence demonstrated in volunteers**. *J Infect Dis* 1995, **171**(2):465-468.
2. Nataro JP, Baldini MM, Kaper JB, Black RE, Bravo N, Levine MM: **Detection of an adherence factor of enteropathogenic *Escherichia coli* with a DNA probe**. *J Infect Dis* 1985, **152**(3):560-565.
3. Joo LM, Macfarlane-Smith LR, Okeke IN: **Error-prone DNA repair system in enteroaggregative *Escherichia coli* identified by subtractive hybridization**. *J Bacteriol* 2007, **189**(10):3793-3803.
4. Mancini J, Weckselblatt B, Chung YK, Durante JC, Andelman S, Glaubman J, Dorff JD, Bhargava S, Lijek RS, Unger KP *et al*: **The heat-resistant agglutinin family includes a novel adhesin from enteroaggregative *Escherichia coli* strain 60A**. *J Bacteriol* 2011, **193**(18):4813-4820.
5. Blattner FR, Plunkett G, 3rd, Bloch CA, Perna NT, Burland V, Riley M, Collado-Vides J, Glasner JD, Rode CK, Mayhew GF *et al*: **The complete genome sequence of Escherichia coli K-12**. *Science* 1997, **277**(5331):1453-1474.
6. Metcalf WW, Jiang W, Wanner BL: **Use of the rep technique for allele replacement to construct new Escherichia coli hosts for maintenance of R6K gamma origin plasmids at different copy numbers**. *Gene* 1994, **138**(1-2):1-7.
7. Bhargava S, Johnson BB, Hwang J, Harris TA, George AS, Muir A, Dorff J, Okeke IN: **The heat resistant agglutinin 1 is an accessory enteroaggregative Escherichia coli colonization factor**. *J Bacteriol* 2009, **191**:4934-4942.

8. Czeczulin JR, Balepur S, Hicks S, Phillips A, Hall R, Kothary MH, Navarro-Garcia F, Nataro JP: **Aggregative adherence fimbria II, a second fimbrial antigen mediating aggregative adherence in enteroaggregative *Escherichia coli***. *Infect Immun* 1997, **65**(10):4135-4145.
9. Simon U, Priefer R, Pühler A: **A broad host range mobilization system for in vivo genetic engineering: transposon mutagenesis in Gram negative bacteria.** *Bio/Technology* 1983, **1**:784-791.
10. Tan MW, Mahajan-Miklos S, Ausubel FM: **Killing of Caenorhabditis elegans by Pseudomonas aeruginosa used to model mammalian bacterial pathogenesis**. *Proc Natl Acad Sci U S A* 1999, **96**(2):715-720.
11. Donnenberg MS, Kaper JB: **Construction of an eae deletion mutant of enteropathogenic Escherichia coli by using a positive-selection suicide vector**. *Infect Immun* 1991, **59**(12):4310-4317.
12. Labar AS, Millman JS, Ruebush E, Opintan JA, Bishar RA, Aboderin AO, Newman MJ, Lamikanra A, Okeke IN: **Regional dissemination of a trimethoprim-resistance gene cassette via a successful transposable element**. *PLoS ONE* 2012, **7**(5):e38142.
13. Ménard R, Sansonetti PJ, Parsot C: **Nonpolar mutagenesis of the ipa genes defines IpaB, IpaC, and IpaD as effectors of Shigella flexneri entry into epithelial cells**. *J Bacteriol* 1993, **175**(18):5899-5906.
