# Supplementary material for: Aggregative Adherence and Intestinal Colonization by Enteroaggregative Escherichia coli Are Produced by Interactions among Multiple Surface Factors
Source: mSphere. 2018 Mar 21;3(2):e00078-18. doi: 10.1128/mSphere.00078-18 (PMC5863034; doi:10.1128/mSphere.00078-18)
Supplement: TABLE S2 [file sph002182496st2.pdf]

Supplemental Table 2. Oligonucleotide primers used in this study.

**Primer**		**Sequence**	**Reference**
PUC18KNcoIF	Amplification of the *apha*-3 cassette and attachment of *Nco*I tails	5'-CATGCCATGGTGACTAAACTAGGAGGAATAAATGGCTAAAATGAGA-3'	This study
PUC18KNcoIR	Amplification of the *apha*-3 cassette and attachment of *Nco*I tails	5'-CATGCCATGGTCATTATTCCCTCCAGGTACTAAAACAATTCATC-3'	This study
Agg1F	Internal to *hra1*	5'-ATTGCGGTTTCAGCGCTTGC-3'	This study
Agg1R	Internal to *hra1*	5'-AGATAGCGATAGCTGAGGTC-3'	This study
AA432F	Internal to aggregative plasmid gene *aatA*	5'-CTGGCGAAAGACTGTATCAT-3'	Cerna et al. 2003 [14]
AA432R	Internal to aggregative plasmid gene *aatA*	5'-CAATGTATAGAAATCCGCTGTT-3'	Cerna et al.

			2003 [14]
aapF	Internal to aggregative plasmid gene *aap*	5’-CTT GGG TAT CAG CCT GAA TG-3’	Cerna et al. 2003 [14]
aapR	Internal to aggregative plasmid gene *aap*	5’-AAC CCA TTC GGT TAG AGC AC-3’-3’	Cerna et al. 2003 [14]
TDOaap1F	Cloning 1 Kb upstream of *aap*	CTT ACT GAA TGA TCG TGT ACT	This study
TDOaap2R	Cloning 1 Kb upstream of *aap*, with *Xho*I tail	CCG CTC GAG TTC ATA TGA CTT CTC TCT AGA TA	This study
TDOaap3F	Cloning 1 Kb downstream of *aap*, with *Xho*I tail	CCG CTC GAG GGT TAA ATA ATA TCT AGC TCT AG	This study
TDOaap4R	Cloning 1 Kb downstream of *aap*	TAC ATC GAT TGT ACA ATT AGA TG	This study
Jdaapf2	Cloning full-length *aap* gene for complementation, with *Sph*I tail	5’-ACA TGC ATG C[1]GT TGT AAT CTG ACA GAT ACC TG-3’	Adapted from Levesque et al.

			[15]
Jdaar2	Cloning full-length *aap* gene for complementation, with *Sph*I tail	5'-ACG CGT CGA C[2]AG AAC CTC GCT TAG ACC TG-3'	Adapted from Levesque et al. [15]
Lev3'XhoI	Amplifying the integron-contained *dfrA7* gene from pASL01a, with *Xho*I tail	5' CCGCTCGAG GGC ATC CAA GCA GCA AG 3'	Adapted from Levesque et al. [15]
Lev5'XhoI	Amplifying the integron-contained *dfrA7* gene from pASL01a, with *Xho*I tail	5' CCGCTCGAG AAG CAG ACT TGA CCT GA 3'	Adapted from Levesque et al. [15]

References

14. Cerna JF, Nataro JP, Estrada-Garcia T: **Multiplex PCR for detection of three plasmid-borne genes of enteroaggregative *Escherichia coli* strains**. *J Clin Microbiol* 2003, **41**(5):2138-2140.
15. Lévesque C, Piché L, Larose C, Roy PH: **PCR mapping of integrons reveals several novel combinations of resistance genes**. *Antimicrobial Agents and Chemotherapy* 1995, **39**(1):185-191.
